# An overview on the green synthesis and removal methods of pyridaben

**DOI:** 10.3389/fchem.2022.975491

**Published:** 2022-07-14

**Authors:** Lingzhu Chen, Mengyuan Pan, Deyu Hu

**Affiliations:** State Key Laboratory Breeding Base of Green Pesticide and Agricultural Bioengineering, Key Laboratory of Green Pesticide and Agricultural Bioengineering, Ministry of Education, Guizhou University, Guiyang, China

**Keywords:** pyridaben, green synthesis, detection rate, household processing, magnetic solid-phase extraction, advanced oxidation processes

## Abstract

Pyridaben is an acaricide widely used around the world to control phytophagous mites, white flies, aphids, and thrips. It is highly toxic to nontarget organisms such as predatory mites, bees, and fishes. Therefore, the occurrence and removal of pyridaben in food and the environment are worthy of concern. This mini-review focuses on pyridaben residue levels in crops, aquatic systems, and soils, as well as the green synthesis and removal of pyridaben. During the period of 2010–2022, pyridaben was reported in monitoring studies on fruits, vegetables, herbs, bee products, aquatic systems, and soils. Vegetable and agricultural soil samples exhibited the highest detection rates and residue levels. One-pot synthesis offers a green chemistry and sustainable alternative for the synthesis of pyridaben. Among traditional home treatments, peeling is the most effective way to remove pyridaben from crops. Magnetic solid-phase extraction technology has emerged as a powerful tool for the adsorption and separation of pyridaben. Photocatalytic methods using TiO_2_ as a catalyst were developed as advanced oxidation processes for the degradation of pyridaben in aqueous solutions. Current gaps in pyridaben removal were proposed to provide future development directions for minimizing the exposure risk of pyridaben residues to human and nontarget organisms.

## Introduction

Pyridaben (2-tert-butyl-5-(4-tert-butylbenzylthio)-4-chloropyridazin-3(2H)-one) is an acaricide containing pyridazinone moiety. It was developed by Nissan Chemical Industries Ltd. in 1980s (Hirata et al., 1995). Over the past decades, pyridaben has become one of the most widely used acaricides (Rand and Clark, 2000; Boulaid et al., 2005; Kim et al., 2015; Chen et al., 2018; Malhat et al., 2021). As many as 408 pyridaben preparations were registered in China by 2022 (Chinese Pesticide Information Network, 2022). A questionnaire in Shaanxi and Shanxi in China showed that pyridaben was one of the most frequently used pesticide in apple orchard, with a used frequency of 86.0% (Shi et al., 2020). As a nonsystematic broad-spectrum acaricide, pyridaben is mainly applied in orchards, crop fields (in particular vegetables), tea plantations, and gardens to control phytophagous mites, white flies, aphids, and thrips (Igarashi and Sakamoto, 1994; Rand and Clark, 2000; Kim et al., 2015). Pyridaben is a powerful inhibitor of glutamate-dependent mitochondrial respiration. Specifically, it inhibits the activity of NADH: ubiquinone oxidoreductase (complex I) and thus inhibits electron transport in insect systems (Hollingworth et al., 1994; Latli et al., 1998).

Pesticides are potentially hazardous to humans and ecosystems. The effects of exposure to pesticides and their transformation products on agricultural commodities consumers and nontarget organisms have been a matter of concern in recent years. In the classification of pesticides by hazard of the World Health Organization, pyridaben is classified as a moderately hazardous (Class II) pesticide (World Health Organization, 2020). The selective inhibition of complex I by pyridaben may constitute part of the pathogenic mechanism of Parkinson’s disease (Gomez et al., 2007). Exposure to pyridaben can induce embryonic loss in early pregnancy in pigs (Bae et al., 2021). Pyridaben is highly toxic to aquatic organisms, such as shrimp (*Mysidopsis bahia*), bluegill sunfish (*Lepornis macrochirus*), rainbow trout (*Oncorhynchus mykiss*), and zebrafish (*Danio rerio*) (Rand and Clark, 2000; European Food Safety Authority, 2010; Ma et al., 2021a). For nontarget arthropods such as honey bees and predatory mites, the hazard quotient values of pyridaben were significantly above the trigger values (European Food Safety Authority, 2010). The results of previous studies have demonstrated that pyridaben had no selectivity to pest mites which are harmful to crops and their natural enemies, predatory mites, which are important for biological control (Hardman et al., 2003; Cheng et al., 2021). The application of pyridaben caused the highest mortality rates on eggs, larvae, and adults of *Euseius scutalis* (Athias-Henriot), and negatively affected the fecundity of adult females among the frequently used acaricides (Döker and Kazak, 2020). Similarly, pyridaben was reported to exhibit high toxicity to phytoseiidae mites such as *Galendromus occidentailis* (Nesbitt), *Neoseiulus womersleyi* (Schicha), *Phytoseiulus persimilis* (Athias-Henriot), and *Neoseiulus cucumeris* (Oudemans) (Alston and Thomson 2004; Park et al., 2011; Cheng et al., 2021). The use of pyridaben should be avoided in integrated pest management programs. Considering the risks of pyridaben to humans and nontarget organisms, its occurrence and removal are worthy of concern.

Xu et al. (2016) have summarized the discovery, synthesis, analysis, pesticide resistance, toxicological mechanisms, and environmental compatibility of pyridaben. European Food Safety Authority has presented overviews on the properties, residues, environmental fate and behavior, and ecotoxicology of pyridaben, and outlined the risk assessment of pyridaben and its metabolites for environmental compartments (European Food Safety Authority, 2010, European Food Safety Authority et al., 2017). The presence and removing methods of pyridaben have not been comprehensively summarized to date. This mini-review focuses on the occurrence of pyridaben which was reported during the period of 2010–2022, as well as the green synthesis and removal of pyridaben.

## Green synthesis of pyridaben

**FIGURE 1:**
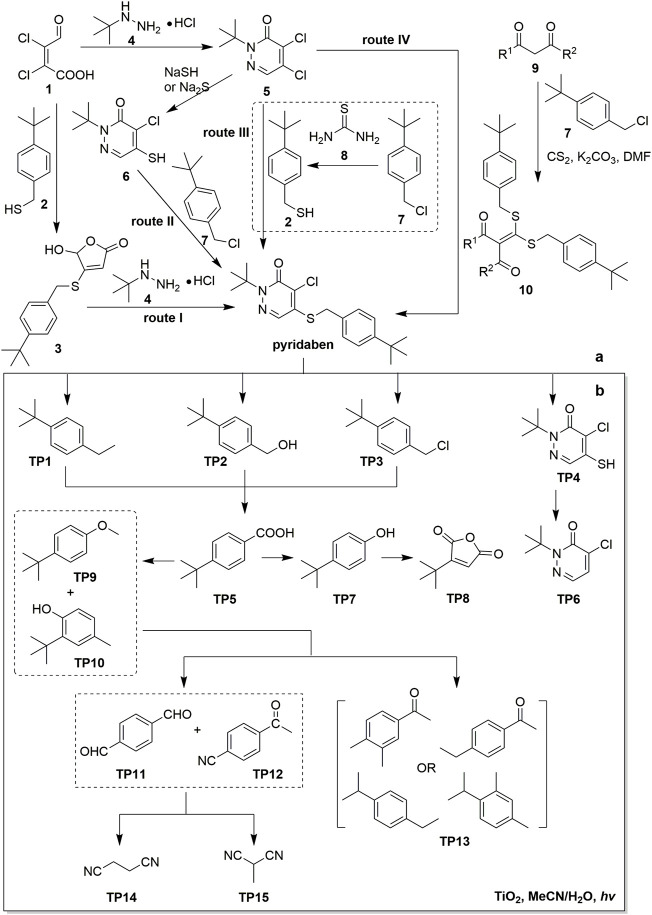
Synthesis and degradation of pyridaben. **(A)** Four major synthetic routes of pyridaben; **(B)** Degradation pathways of pyridaben in photocatalytic systems.

To date, four major routes for the synthesis of pyridaben have been reported (Figure 1A) (Shogo et al., 1995; Xu et al., 2016; Dang et al., 2020). Route I which involves a key furanone intermediate **3** has no practical value due to its low yield. In route II and other similar synthetic routes, the use of NaSH or NaS_2_ inevitably results in the formation of irritant and toxic gas H_2_S, which poses risks to operators and environments. Route III is the thioetherification between dichloropyridazinone **5** and *p*-(tert-butyl)benzyl mercaptan **2**. For the synthesis of **2**, thiourea **8** is an alternative of thiolation reagent NaSH. The reaction between **5, 7**, and **8** can be performed in one pot, which is more convenient and efficient than stepwise reactions. Route III is a green synthetic route because it gives pyridaben in high yield and avoids the generation of toxic gas or undesired sulfide by-products. Route IV uses *α*-oxo ketene dithioacetal **10** instead of *p*-tert-butyl benzyl mercaptan **2** to condense with **5**. This route avoids the use of NaSH, but high boiling point solvents such as *N*,*N*-dimethylformamide or dimethyl sulfoxide is required for the synthesis of **10**.

## Occurrence of pyridaben

Pesticide residues in crops and the surrounding environment are inevitable after application. The issue of pesticide residues has aroused public awareness and government concerns. According to the criteria presented by Andreu and Picό (2012), pyridaben is susceptible to accumulating in biota due to its low water solubility (0.022 mg/L at 20°C) and high octanol-water partition coefficient (log Kow = 6.37 at 23°C) (European Food Safety Authority, 2010). As a result, pyridaben and its transformation products have the potential to accumulate in human bodies and ecosystems.

### Pyridaben levels on agricultural products

Pyridaben is widely applied on fruit trees and vegetables to control red spider mites (*Tetranychus cinnbarinus*) and flea beetles (*Phyllotreta spp*). The results of the 6th Chinese total diet study indicated that pyridaben was the most frequently detected acaricides with a detection rate of 11.8% (34/288) (Xin et al., 2020). Among the 12 kinds of dietary samples, pyridaben was mainly detected in vegetable and fruit samples, with the maximum concentration of 49.55 μg/kg. A three-year (2013–2015) monitoring survey of pesticide residues on peaches in China found pyridaben residues in 10.6% of samples (Li et al., 2018). The residue values ranged from 0.01 to 0.28 mg/kg, which was lower than the maximum residual level (MRL) value of European Union (0.3 mg/kg). In another study of five local fruit cultivars in Shanghai, 1 out of 40 peach samples was positive for pyridaben, with a residue value of 7.8 μg/kg (Zhang et al., 2021). Pesticide residues in 2922 mandarin, tangerine, and orange samples from China were evaluated during the period of 2013–2018 (Li et al., 2020). Pyridaben was detected in 7.2% samples, and the residue levels were in the range of 0.005–0.63 mg/kg, which were lower than the MRL of China (2 mg/kg). The cumulative chronic dietary intakes did not pose health risks for Chinese general population and children. Ping et al. (2022) analyzed the residues of 24 pesticides in 293 greenhouse vegetable samples grown under modern urban agriculture from Beijing. Pyridaben was detected in pakchoi and tomato samples, with detection rates of 18.8% and 2%, respectively. The concentration levels of pyridaben residue in pakchoi ranged from 130.0 to 3400.2 μg/kg, which were the highest among the residues of 24 investigated pesticides; they accounted for 42.7% to the estimated daily intake. Fortunately, the hazard index was far lower than 1, which indicated low dietary risk. In another study, 19,786 vegetable samples collected from Xinjiang Uygur Autonomous Region of China during the period of 2010–2014 were analyzed (Wu et al., 2017). Pyridaben was found in 645 samples, 14 of which exceeded the MRL values set by the Chinese government. The residues of pyridaben contributed to 7.0% of the hazard indices through consuming vegetables.

In 91 snow fungus samples collected from Fujian Province, pyridaben was one of the highest frequently detected pesticide, with a detection rate of 9.9% and residues in the range of 0.011–0.046 mg/kg (Yao et al., 2020). In 151 honeysuckle samples collected from different planting bases in China, pyridaben was detected in 42.19% samples in 2017 and 45.98% samples in 2018, with residues in the range of 0.001–0.075 mg/kg and 0.001–0.080 mg/kg, respectively. The short-term, long-term, and the cumulative risks of adults to pesticide in honeysuckle were all negligible (Wu et al., 2021).

Pyridaben exhibited high toxicity to bees (LD_50_ = 0.535 μg/bee for acute oral toxicity and LD_50_ = 0.024 μg/bee for acute contact toxicity) (European Food Safety Authority, 2010; Bahreini et al., 2020). As one of the most lipophilic pesticides (log Kow = 6.37), pyridaben are easily accumulated in wax given the lipophilic nature of beeswax (Wilmart et al., 2021). In the survey from spring 2016 to autumn 2017, pyridaben was detected in 2.1% pollen samples (4/189) and 9.7% beebread samples (22/226) collected from five major beekeeping areas in China (Tong et al., 2018). The detection rate in pollen was smaller than that in beebread, but the residues in pollen were much higher than that in beebread. The mean values of pyridaben residue in pollen and beebread were 37.4 and 10.4 ng/g, respectively. In another investigation during spring of 2016 to four different types of bee wax samples collected from apiaries in Belgian, 1 out 182 of brood comb wax samples was positive for pyridaben, with a residue value equal to 0.01 mg/kg (Agrebi et al., 2020).

### Pyridaben levels in environmental matrices

At the beginning of autumn, pyridaben is often used to prevent European red mite in apple orchards in China. The surface soil samples collected from an apple orchard of China in August 2017 were analyzed (Shi et al., 2020). It is stated that 100% of soil samples were positive for pyridaben, with concentrations ranging from 1.6 to 47.2 ng/g dw. In a study of the orchards and vegetable field in southern China, pyridaben was detected in collected agricultural soils with a detection rate of about 6% (Pan et al., 2019). In the soil monitoring project in five districts of southern Jordan during 2016 and 2017, pyridaben was found with high frequency and high concentration (12%, up to 5,820 μg/kg) (Kailani et al., 2021). Pyridaben is characterized by low mobility in soil and high concentration levels in soil due to it low solubility in water. (Sabzevari and Hofman, 2022). In turn, its penetration to ground water and surface water is limited. The detection frequency of pyridaben was relatively low in sediment-river-water systems, sediment-seawater system, and surface water from tropical river basins in China (Zheng et al., 2016; Tan et al., 2021; Wang et al., 2022). A three-year monitoring program of Abou Ali River located in the north of Lebanon showed that the detection frequency of pyridaben in surface water and groundwater were 18% and 20%, respectively, with a maximum concentration of 0.49 μg/L (Jabali et al., 2020).

## Removal of pyridaben residues

Pyridaben residues in agricultural commodities and environmental matrices would lead to hazardous effects on human and nontarget organisms. Various methods are employed to remove pyridaben for minimizing the risk of human and ecosystems exposure to it.

### Household processing

Household processing is the most common method to reduce the risk of pesticides for agricultural commodities consumers. The effects of food processing on pesticide residue levels can be evaluated by the processing factor (PF) calculated by Eq. 1 (Food and Agriculture Organization and World Health Organization, 2006):

$$\text{PF}=\;\frac{residues\;in\;processed\;food\;\left(mg/kg\right)}{residues\;in\;raw\;materials\;\left(mg/kg\right)}$$ (1)

Table 1 summarizes the effect of various household processing, including washing, blanching/boiling, frying/drying, peeling, and juicing on pyridaben residues.

**TABLE 1:** Effect of house processing techniques on pyridaben residues.

Household processing condition	Agricultural commodity	Reduction ratio (%)	Processing factor (PF)	References
Washing	Hot pepper fruit	42.5–54.0	0.46 –0.57	Kim et al. (2015)
	Apple	5.71	0.94	Han et al. (2014)
	Tomato		0.90	Boulaid et al. (2005)
	Green bean		0.32	Aguilera et al. (2014)
	Apricot	19.5		Cámara et al. (2020)
	Cowpea		1.00	Huan et al. (2015)
Blanching/boiling	Hot pepper fruit (after washing)	70.1–83.5	0.16–0.30	Kim et al. (2015)
	Cowpea		1.06–1.09	Huan et al. (2015)
	Tomato		2.1	Boulaid et al. (2005)
	Green bean		0.67	Aguilera et al. (2014)
Frying/drying	Hot pepper fruit (after washing and blanching)	41.7–72.6	0.27–0.58	Kim et al. (2015)
	Kiwifruit		1.05	Wang et al. (2020)
	Honeysuckle		7.2–11.2	Xiao et al. (2022)
	Cowpea		0–0.14	Huan et al. (2015)
Peeling	Kiwifruit	70.5–97.84	0.15	Wang et al. (2020)
	Apple	80.3	0.12	Han et al. (2014)
	Tomato	70	0.3	Boulaid et al. (2005)
Juicing	Kiwifruit	42.0–53.2	0.51	Wang et al. (2020)
	Apple	66.7	0.22	Han et al. (2014)

#### Washing

In most cases, washing is the first step in household treatment for food to remove dust and pesticide on the surface. Data reported in the previous studies about the effect of washing on pyridaben residues are contradictory. Washing can substantially reduce residue levels of pyridaben in hot pepper fruits and green bean pods, with PF of 0.46–0.57 and 0.32, respectively (Aguilera et al., 2014; Kim et al., 2015). On the contrary, the application of an intensive washing cannot reduce the residue levels of pyridaben in tomatoes, apples, and cowpeas (Boulaid et al., 2005; Han et al., 2014; Huan et al., 2015). In the processing of apricots, pyridaben residues were reduced with a loss of 19.5% (Cámara et al., 2020). The efficiency of washing of a pesticide is dependent on many factors, such as the washing procedure and physicochemical properties of the pesticide and the crop epidermis. As a nonsystemic pesticide, pyridaben cannot be transported easily into the inner part of crops and thus mainly remains on the surface. It has been demonstrated that no correlation exists between water solubility of pesticides and pesticide removal by washing (Christensen et al., 2003; Chung, 2018). The decisive factor is the amount of pyridaben dissolved in the waxy layer, which is related to the nature of cuticle. In most cases, pyridaben is strongly retained by waxes of fruits and vegetables due to its high liposolubility. Therefore, washing is relatively inefficient in removing pyridaben residues.

#### Blanching/boiling

Blanching or boiling is a traditional method of treating vegetables to make them edible. The efficiency of blanching/boiling is affected by various factors such as cooking time, temperature, moisture loss, and pesticide degradation. Kim et al. (2015) found that blanching after washing was the most efficient method to reduce the level of pyridaben residues in pepper fruit. Compared with washing, blanching improved the removal of pyridaben by 27.6%–29.5%. The increase in the reduction ratio can be attributed by the squeezing step in blanching, the purpose of which was to remove excess water. In another study of green bean pod treatment, the PF for pyridaben was 0.67 (Aguilera et al., 2014). As reported by European Food Safety Authority et al. (2017), pyridaben is stable under the treatment of pasteurization, baking/brewing/boiling, and sterilization. The degradation or hydrolysis of pyridaben appears to not occur during the boiling process. Meanwhile, pyridaben has low volatility (1.18 × 10^–6^ mmHg, 20°C). The concentration effect was observed for the treatment of cowpea and tomato due to water loss, with PF of 1.6–1.11 and 2.1, respectively (Boulaid et al., 2005; Huan et al., 2015).

#### Frying/drying

Frying/drying food with oven or frying pan is a traditional method of food treatment. On the one hand, the pesticide residues may decrease due to thermal degradation. On the other hand, pesticide residues can be concentrated as a result of dehydration. For example, frying and stir-frying were effective to reduce pyridaben residues on cowpeas with PF = 0–0.14 (Huan et al., 2015). Compared with boiling, the temperature of frying and stir-frying was higher and the residues of pyridaben were readily transferred from cowpea into oil due to its high liposolubility. Consistently, the residue level of pyridaben in fried oil increased as frying time progressed. The results of another study indicated that frying can reduce the pyridaben residues in hot pepper fruit, but the effect was inconsiderable compared with that of washing even with increased temperature and prolonged time (Kim et al., 2015). On the contrary, drying significantly increased pyridaben residues in kiwifruits and honeysuckles (Wang et al., 2020; Xiao et al., 2022).

#### Peeling

Peeling is commonly the first step in treatment of fruits and certain vegetables. Pyridaben primarily deposits on the peel of the edible part of crops due to its nonsystemic characteristic. In general, peeling is the most efficient method to reduce pyridaben residues. For example, peeling significantly affected the removal of pyridaben from kiwifruits and tomatoes, with PF of 0.15 and 0.3, respectively (Boulaid et al., 2005; Wang et al., 2020). Similarly, peeling and coring significantly affected the reduction of pyridaben on apples (PF = 0.12) (Han et al., 2014). The cuticular wax of apples served as a transport barrier.

#### Juicing

Juicing is one of the most common household processes in fruit treatment. The removal efficiency of juicing is determined by the sort of fruit. After peeling, pyridaben residues in the flesh of kiwifruit were concentrated by juicing due to evaporation of water (Wang et al., 2020). Nevertheless, the pyridaben residues in apple can be effectively removed by juicing, with a PF of 0.22, which was attributed to the removal of apple pomace in the juicing process of apple (Han et al., 2014).

### Magnetic solid–phase extraction

In the recent decades, magnetic solid–phase extraction (MSPE) has emerged as an effective tool for enrichment and separation of pesticide residues (Yu et al., 2022; Zhao et al., 2022). After adsorption, the separation of adsorbent and sample solutions can be easily achieved by applying an external magnetic field. MSPE technology combines the high efficiency of solid-phase extraction and the convenience of magnetic separation. Fe_3_O_4_/MoS_2_@G nanocomposite was prepared *via* a co-mixing solvothermal method and employed for the MSPE of pyridaben in an emulsified aqueous solution using dichloromethane as an extractant (Ma et al., 2021b). As an amphiphilic nanocomposite, Fe_3_O_4_/MoS_2_@G consists of hydrophilic and hydrophobic moieties. The π-π stacking interaction between Fe_3_O_4_/MoS_2_@G and the π electronic system of pyridaben was responsible for the effective adsorption. The extraction recovery for pyridaben was greater than 87% after reusing for 9 consecutive cycles. Zhao et al. (2022) reported a metal–organic framework material as the absorbent of pyridaben. In this study, NiO/Co@C magnetic nanocomposites were assembled as a MSPE adsorbent. NiO/Co@C contained a hollow porous structure, resulting in high pyridaben adsorption performance, which was up to 51.3 mg/g. The extraction recovery loss of pyridaben was less than 10% after 9 cycles.

### Advanced oxidation processes

Advanced oxidation processes (AOPs) were also employed as a powerful way to degrade pesticides (Zeshan et al., 2022). The catalyzed efficiency of TiO_2_ particles in acetonitrile/water solutions with irradiation of medium pressure Mercury lamp were investigated (Zhu et al., 2004; Zhu et al., 2005). The degradation kinetics followed the Langmuir-Hinshelwood model with kinetic constant k = 4.3 × 10^–5^ mol/L min and equilibrium adsorption constant K = 3.1 × 10^3^ L/mol (Zhu et al., 2004). The degradation rate of pyridaben increased with the increase of catalyst TiO_2_, nevertheless, an overdose of TiO_2_ would retard the photocatalytic reaction (Zhu et al., 2005). Direct photolysis dominated the photodegradation of pyridaben at 300 < λ ≤ 360 nm, while photocatalysis degradation by TiO_2_ was dominant at λ ≥ 360 nm. In TiO_2_ aqueous dispersions, cationic surfactant cetyltrimethyl ammonium bromide can help pyridaben coadsorb on TiO_2_ particles surface and facilitate the photocatalytic degradation (Zhu et al., 2007). A low dose of H_2_O_2_ can also improve the photocatalytic rate by removing surface-trapped electrons and increasing the efficiency of hole utilization on TiO_2_ particles, as well as providing sufficient oxygen for photocatalytic degradation. **TP1–TP15** were identified as photolytic products using gas chromatography tandem mass spectrometry (Figure 1B) (Zhu et al., 2004; Zhu et al., 2005). The primary reaction intermediates **TP1–TP4** underwent subsequent oxidation and rearrangement steps, which led to the formation of various short-chain compounds. Mechanism studies suggested that the key step in the photodegradation of pyridaben was the cleavage of C–S bond between benzyl and pyridazinone group.

## Conclusion and perspectives

This mini-review presents the levels of pyridaben in agricultural products, water and soil reported between 2010 and 2022. The removal methods of pyridaben are also summarized, including household processing, MSPE, and AOPs technologies. The one-pot reaction of dichloropyridazinone, *p*-tert-butylbenzyl chloride, and thiourea is a green synthesis method of pyridaben. As an acaricide used worldwide, pyridaben has been reported in monitoring studies on fruits, vegetables, herbs, and bee products. In general, vegetable samples exhibit the highest detection rates and residue levels. Pyridaben has low solubility in water and easily remains in soil. It exhibits high concentration levels and detection rates in agricultural soil samples. Peeling is the most effective household method for removing pyridaben residues, while washing, blanching/boiling, frying/drying, and juicing show limited removal efficiencies and even show concentration effects. Notably, some vegetables, such as leafy vegetables, do not have peelable outer layers. Therefore, effective vegetables processing methods need to be developed. MSPE and AOPs technologies have been developed for the removal of pyridaben. These methods are effective in treating pyridaben in aqueous solutions. However, effective soil treatment methods have not been developed so far. This gap deserves further concern given the tendency of pyridaben to remain in soil.
